# Family-centered rounds and medical student performance on the NBME pediatrics subject (shelf) examination: a retrospective cohort study

**DOI:** 10.3402/meo.v21.30919

**Published:** 2016-04-15

**Authors:** Tiffany N. Kimbrough, Victor Heh, N. Romesh Wijesooriya, Michael S. Ryan

**Affiliations:** 1 Department of Pediatrics, Virginia Commonwealth University, Richmond, VA, USA; 2 CORE Research Office, Heritage College of Osteopathic Medicine, Ohio University, Dublin, OH, USA

**Keywords:** family-centered rounds, NBME subject examination, undergraduate medical education

## Abstract

**Objective:**

To determine the association between family-centered rounds (FCR) and medical student knowledge acquisition as assessed by the National Board of Medical Examiners (NBME) pediatric subject (shelf) exam.

**Methods:**

A retrospective cohort study was conducted of third-year medical students who graduated from Virginia Commonwealth University School of Medicine between 2009 and 2014. This timeframe represented the transition from ‘traditional’ rounds to FCR on the pediatric inpatient unit. Data collected included demographics, United States Medical Licensing Examination (USMLE) Step 1 and 2 scores, and NBME subject examinations in pediatrics (PSE), medicine (MSE), and surgery (SSE).

**Results:**

Eight hundred and sixteen participants were included in the analysis. Student performance on the PSE could not be statistically differentiated from performance on the MSE for any year except 2011 (*z*-score=−0.17, *p*=0.02). Average scores on PSE for years 2009, 2010, 2013, and 2014 were significantly higher than for SSE, but not significantly different for all other years. The PSE was highly correlated with USMLE Step 1 and Step 2 examinations (correlation range 0.56–0.77) for all years.

**Conclusions:**

Our results showed no difference in PSE performance during a time in which our institution transitioned to FCR. These findings should be reassuring for students, attending physicians, and medical educators.

**What This Study Adds**: Learners have previously expressed concern over medical knowledge acquisition during family-centered rounds. This study demonstrated no detrimental effect on knowledge acquisition during a period in which family-centered rounds was introduced.

Since its introduction in the early 2000s, family-centered rounds (FCR) has emerged as the preferred method for rounding in pediatric settings across the United States (1). Defined broadly as ‘interdisciplinary rounds at the bedside in which the patient and family share in the control of the management plan as well as in the evaluation process itself’ (2), a focus on family-centered care (3–5) and the development of FCRs (3, 4) have received endorsements through the American Academy of Pediatrics (3) the Institute of Medicine (4) as well as the Accreditation Council for Graduate Medical Education (5).

FCR has been shown to benefit families who report better understanding of the treatment plan, increased involvement in care, and more consistent communication with hospital staff (1, 6, 7). Despite its advantages for patients and families, there have been concerns expressed over the potential impact this rounding style may have on education. Specifically, there is a body of evidence citing resident and student perceptions of decreased ‘didactic’ teaching (8), increased discomfort asking specific management questions, and limited time to discuss management options (9–11). The literature describing the association between FCR and teaching has been limited to assessments of learners’ perceptions without data to objectively address the relationship between FCR and medical knowledge acquisition.

The purpose of this study was to assess the association between FCR and medical student knowledge acquisition during the pediatrics clerkship, using the National Board of Medical Examiners pediatric shelf exam (NBME PSE) as a validated and objective marker of knowledge attainment.

## Methods

This was a retrospective cohort study of third year medical students who graduated from the Virginia Commonwealth University School of Medicine (VCUSOM) – Richmond campus between 2009 and 2014. VCUSOM is a large, public school of medicine associated with an urban university in Richmond, VA. During the study period, the majority of students (approximately 85%) were assigned to the Richmond campus for their clinical years while a minority completes their experience at the Fairfax/INOVA campus in Fairfax, VA. All Richmond campus students rotated through an 8-week pediatrics core clerkship, which included 4 weeks of outpatient/nursery rotations and 4 weeks of inpatient rotations. The Children's Hospital of Richmond is a children's hospital within a hospital, located on the VCUSOM campus and served as the primary teaching site for the inpatient rotations throughout the study period.

### Implementation of FCR

In 2008, our institution created an FCR committee to explore the development and integration of FCR into our rounding model on the inpatient general pediatrics service. We based our system of FCR on the model previously described by Muething et al. (12). FCR at our institution involved bedside rounds with the attending, resident physicians, medical students, nursing staff, and other ancillary services in attendance. Families were educated about FCR at admission to the hospital. The student or intern assigned to the patient presented after introducing members of the team and eliciting concerns or questions from the family and/or the patient. Family members were active participants in the development of the plan, including management decisions and discharge goals.

Throughout the study period, the general pediatrics service was comprised of two teams, each containing one generalist attending, one third-year resident, one second-year resident, two–three interns, and three–four medical students. Twenty-five to thirty medical students rotated through the clerkship at a given time, and each completed a 2-week rotation on the inpatient general pediatrics service. Students were expected to carry and present a minimum of two general pediatric patients at all times. Attending physicians on the general pediatric service were mostly members of the pediatric hospitalist medicine division, with a subset of general academic pediatricians and subspecialty faculty.

We studied the relationship between FCR and student performance for VCUSOM Class of 2009–2014 to represent our transition from traditional rounds to full integration of FCR. Prior to the Class of 2010, no attending physicians or residents utilized FCR. Beginning with the Class of 2010, faculty and resident development opportunities were designed to familiarize and share best practices regarding FCR. In each consecutive year beginning with the Class of 2010 and moving forward to Class of 2013, adoption of the FCR model was embraced by a variable, but continually increasing number of attending physicians. Beginning with the Class of 2014, all attending physicians providing care on the general pediatrics service were required to conduct FCR. Additionally at that time, all attending physicians, residents and students were given an overview of the process and expectations for their roles in FCR.

### Design

Participants were identified through an internal database of medical student records maintained through the VCUSOM curriculum office. Data were collected for each medical student including demographics, United States Medical Licensing Examination (USMLE) Step 1 and 2 scores, and performance on the NBME subject examinations in pediatrics (PSE), medicine (MSE), and surgery (SSE). The USMLE Step 1 and 2 examinations were selected to represent standardized objective, pre- (USMLE Step 1) and post- (USMLE Step 2) clerkship assessments of knowledge acquisition. The medicine and surgery subject examinations were selected to compare the same student cohort performance on other standardized examinations during the core clerkships, which did not incorporate FCRs.

Participants were included in the analysis if they had complete academic records available for analysis. Participants were excluded if they 1) completed any portion of their core clerkship at the Fairfax/INOVA campus, 2) had incomplete data, or 3) completed any of their core clerkships outside of the traditionally structured third year.

We converted scores from each examination to standardized *z*-scores. The purpose of this conversion was twofold: 1) it allowed us to compare between examinations that utilize a different raw score (e.g., the USMLE uses a three-digit score, while the NBME subject examinations use a two-digit score), and 2) it allowed us to control for national trends of mean scores over the study period.

### Data analysis

*Mean* and *standard deviation* were used to describe distribution of scores. *Pearson correlation* was used to determine relationship between two tests. Two inferential statistical techniques, *one-sample z-test* and *paired t-test*, were used to compare average scores 1) to national average and 2) between any two-subject shelf exams. *Alpha* of 0.05, *two-tailed* was used to determine evidence of statistical significance and *R-squared* (square of correlation coefficient) for evidence of practical significance. All statistical analyses were performed using SPSS version 22. The study qualified for exemption by the Virginia Commonwealth University Institutional Review Board.

## Results

A total of 860 Richmond-based participants were identified through a review of the internal database. Forty-eight were excluded due to incomplete data and/or completion of core clerkship(s) outside the traditionally structured third year. This resulted in 816 who were included in the analysis. Participants were similar in terms of gender and ethnicity over the study period. Mean Step 1 and Step 2 scores increased parallel to national trends. A summary of the demographic variables is presented in Table 1.

**Table 1:** Demographic data for VCUSOM students

		Demographics			Sample mean scores (SD)				
		
Class year	FCR adoption phase	n	% Female	% White	Step 1	Step 2	PSE	MSE	SSE
2009	No FCR	124	42	57	218 (20)	228 (24)	74.8 (8.5)	76.0 (7.6)	70.5 (7.2)
2010	Variable	134	49	57	223 (20)	233 (23)	78.0 (7.6)	78.1 (7.3)	73.1 (8.3)
2011	Variable	130	44	65	225 (21)	236 (20)	77.3 (8.1)	78.3 (7.5)	74.2 (9.1)
2012	Variable	143	46	62	225 (21)	240 (20)	78.7 (8.7)	79.6 (7.6)	75.5 (8.2)
2013	Variable	143	48	56	225 (20)	239 (19)	80.0 (8.7)	79.4 (7.6)	75.5 (8.8)
2014	Complete	138	40	54	231 (19)	242 (17)	79.2 (8.3)	80.7 (8.9)	75.6 (9.3)

PSE, pediatric subject examination; MSE, medicine subject examination; SSE, surgery subject examination; VCUSOM, Virginia Commonwealth University School of Medicine; SD, standard deviation.

### Performance on the pediatrics shelf exam

Overall, medical students’ performance on the PSE was at or above the national average for each year in the study period, including both transitional and consistent adoption of FCR implementation. For Class of 2009, 2011, and 2012, students’ performance were not statistically different from national average (*p>*0.05), whereas for Class of 2010, 2013, and 2014, students performed significantly above national average (*p<*0.05), achieving 0.17–0.31 standard deviations units above national average (Table 2).

**Table 2:** VCUSOM student performance on pediatric shelf exam (PSE) versus national average

		Performance on PSE		Analysis			
		
Class year	FCR adoption phase	Sample mean (SD)	National mean (SD)	*z*-score difference	*z*-test, two-tailed	*p*	Conclusion
2009	No FCR	74.8 (8.5)	74.7 (8.1)	0.01	0.16	0.87	At national average
2010	Variable	78.0 (7.6)	75.8 (8.0)	0.27	3.29	<0.01	Above national average
2011	Variable	77.3 (8.1)	76.2 (8.7)	0.12	1.42	0.15	At national average
2012	Variable	78.7 (8.7)	77.4 (8.7)	0.14	1.78	0.08	At national average
2013	Variable	80.0 (8.7)	77.3 (8.7)	0.31	3.75	<0.001	Above national average
2014	Complete	79.2 (8.2)	77.7 (8.8)	0.17	2.06	0.04	Above national average

VCUSOM, Virginia Commonwealth University School of Medicine; SD, standard deviation.

### Comparison with other exams

Student performance on PSE was compared with MSE and SSE for each class year. Results showed that, overall, students’ performance on the PSE was not significantly different from their performance on MSE except for Class of 2011, whose performance on MSE was significantly higher than on PSE (*p=*0.02). However, the average *z*-score difference between PSE and MSE for Class of 2011 was only 0.17 standard deviation units. In addition, students scored significantly higher on PSE than on the SSE for 4 of the 6 years under review. Differences in performance were as high as 0.15–0.26 standard deviation units in favor of PSE (Table 3).

**Table 3:** VCUSOM student performance on NBME pediatric versus medicine and surgery subject examinations

		Pediatric versus medicine subject exam				Pediatric versus surgery subject exam			
		
Class year	FCR adoption phase	Mean difference PSE versus MSE *z*-score	*t*-test	*p*	Correlation coefficient	Mean difference PSE versus SSE *z*-scores	*t*-test	*p*	Correlation coefficient
2009	No FCR	0.01	0.12	0.91	0.45***	0.16**	2.69	0.01	0.77***
2010	Variable	0.02	0.29	0.77	0.57***	0.19**	2.45	0.01	0.56***
2011	Variable	–0.17*	–2.47	0.02	0.63***	0.01	0.10	0.92	0.59***
2012	Variable	–0.13	–1.76	0.08	0.58***	0.10	1.48	0.14	0.62***
2013	Variable	0.10	1.34	0.18	0.60***	0.26***	3.61	<0.001	0.61***
2014	Complete	–0.10	–1.41	0.16	0.62***	0.15*	2.45	0.02	0.72***

VCUSOM, Virginia Commonwealth University School of Medicine; NBME, National Board of Medical Examiners; PSE, pediatric subject examination; MSE, medicine subject examination; SSE, surgery subject examination.

*** *p*≤0.001

** *p*≤0.01

* *p*≤0.05.

**Fig. 1:**
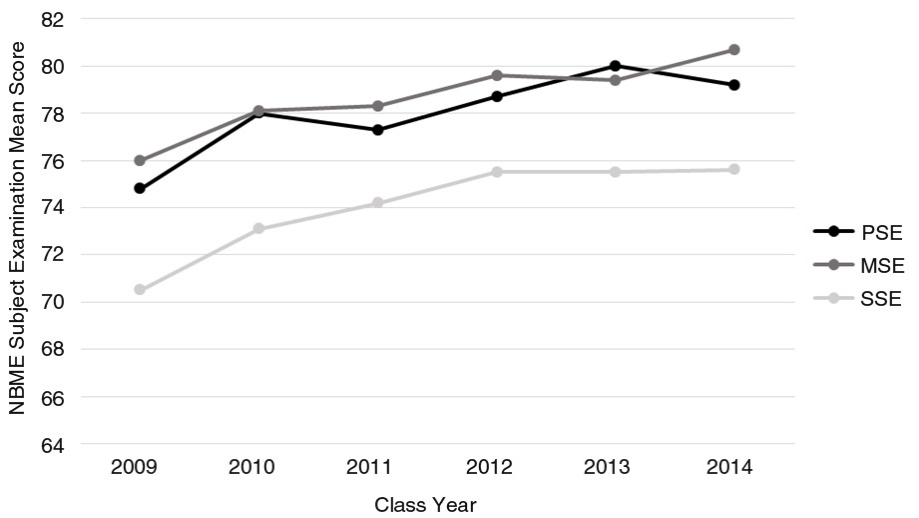
NBME subject examination scores over time. The NBME scores are shown for the pediatric, medicine, and surgery subject examinations (PSE, MSE, and SSE) for the classes of medical students we studied, Class of 2009–2014, while our institution implemented family-centered rounds in inpatient pediatrics.

PSE was highly correlated with both MSE and SSE. Correlations between PSE and MSE during transitional and consistent adoption of FCR implementation were significant, *p*<0.001, with coefficients that ranged from 0.45 to 0.62. Highly significant correlations were also obtained between PSE and SSE with coefficients of 0.56–0.72, *p*<0.001 (Table 3). Figure 1 demonstrates the relationship between these examinations from year to year.

The PSE was also highly correlated with USMLE Step 1 and 2. Correlations between PSE and USMLE 1 for each year ranged from 0.56 to 0.71 and proportion of variance in USMLE 1 explained by PSE ranged from 31 to 50%. PSE also significantly predicted USMLE 2; correlation coefficients ranged from 0.61 to 0.77, *p*<0.001, and proportion of variance explained ranged from 38 to 60% (Table 4).

**Table 4:** NBME pediatric subject examination as a predictor of USMLE Step 1 and 2

		USMLE Step 1		USMLE Step 2	
		
Class year	FCR adoption phase	Correlation	*R*-squared (%)^a^	Correlation	*R*-squared (%)^b^
2009	No FCR	0.60***	35	0.61***	38
2010	Variable	0.56***	31	0.67***	45
2011	Variable	0.63***	40	0.69***	47
2012	Variable	0.61***	37	0.69***	48
2013	Variable	0.62***	38	0.68***	46
2014	Complete	0.71***	50	0.77***	60

NBME, National Board of Medical Examiners; USMLE, United States Medical Licensing Examination.

*** *p*≤0.001

** *p*≤0.01

* *p*≤0.05.

a *R*-squared denotes practical significance; overall correlation across all cohorts is 0.63*** with 39% proportion of variance explained.

b *R*-squared denotes practical significance; overall correlation across all cohorts is 0.68*** with 47% proportion of variance explained.

## Discussion

Our results showed no significant difference in performance on the NBME PSE as we implemented FCR in our institution. Throughout the study period, students performed at or better than the national mean on the NBME PSE and comparable on the NBME subject examinations in surgery and medicine, which utilizes traditional rounding formats. While other studies have assessed the impact of FCR on various aspects of medical education, this is the first to objectively examine the association between FCR and medical knowledge acquisition.

### Educational outcomes of FCR

Bedside teaching has been studied extensively in medical education literature (13–16) with clear benefits on interpersonal communication, professionalism, and physical exam skill development in trainees (13, 15). Growing literature suggests that FCR, as a specific method of bedside teaching, promotes trainee growth in attitudes (17), communication skills (1, 18), and physical examination skills (1) through role modeling opportunities and feedback (1, 19). While there are readily apparent benefits to FCR, the impact on medical knowledge acquisition is less clear and often thought of as a trade-off to the practice.

### Medical knowledge acquisition

FCR is often criticized for promoting anxiety among trainees (15, 17, 20) and increasing the time spent on rounds (21). It has been suggested that the anxiety created by this rounding method and the time spent away from formal ‘didactic learning (8)’ may serve as a detriment to trainee education. However, the results of this study and others suggest that knowledge acquisition may be preserved or even enhanced indirectly from the time spent *preparing* for FCR. In a recent qualitative study, the prospect of presenting in front of families was shown to motivate students to engage in more independent reading (18). Self-directed learning is an active learning approach shown to facilitate deep learning (22). Families presented a natural inspiration to ‘read more’ in an attempt to appear confident and competent on rounds (18).

The benefit of anxiety in learning is consistent with the educational literature. Either too little or too much anxiety inhibits learning while a reasonable level serves to optimize the experience (23). Similarly, FCR serves as a setting to *apply* knowledge to practice, which is considered a higher-order educational outcome (24). We would therefore suggest that the prospect of presenting in front of families and the time spent in the experience may, in reality, serve as a strong incentive to stimulate self-directed learning opportunities. Such benefits may be indirect in nature, but perhaps more beneficial for knowledge acquisition than time spent in the classroom or conference room.

### Validity of the NBME subject examination

We selected the NBME subject examinations as an outcome measure due to literature and experience utilizing the test as an objective measure of medical knowledge. The vast majority of clerkships currently utilize the NBME subject examinations (25) because they are ‘highly reliable exam(s) that can provide a measure of knowledge’ (26). Validity evidence includes convergent relationships between the subject examinations, USMLE Step 1, 2, and 3 (27–30), residency-specific in training examinations (31–33) and board passage rates (34).

There are also data to suggest that the clerkship experience can have an impact on performance on the NBME subject examinations. For example, the length of time on a given clerkship has been shown to impact performance on the subject examinations in surgery, medicine, and OB/GYN (35–37). Similarly, innovative educational strategies have been shown to increase subject examination performance (38, 39). Therefore, the data would suggest that viewed as an educational intervention, FCR could have an impact on subject examination. The conclusion that no difference was seen should be, at a minimum reassuring, particularly given the previously described benefits of the practice.

### Limitations

There are several limitations to this study. First, the study was retrospective in nature thus limiting our ability to assess the quality of FCR among individual faculty members. Additionally, medical students at VCUSOM spent only half of their time on the pediatric clerkship in the inpatient setting, allowing for opportunities to gain pediatric knowledge in other arenas. Next, we excluded from analysis those students who interrupted their training. It may be that by excluding those students, we in effect eliminated those who may have been more affected by FCR than the traditional student. However, these students made up a small fraction of the total student body matriculating through VCUSOM thus limiting the overall impact on the results. Another consideration may include annual revisions to core instruction (e.g., lectures and small group learning experiences) during the clerkship. Such changes are part of the continuous quality improvement cycle of the curriculum and may represent a confounder to the analysis of our data.

Finally, it is possible the NBME PSE did not serve as an adequate marker for knowledge gained on inpatient wards. The NBME subject examination did not allow for segregation of ‘inpatient’ from ‘outpatient’ material or allow for breakdown of student scores. An ideal outcome measure would have been a validated instrument designed to assess inpatient-specific knowledge; however, no such tool exists at present. Thus, we felt the NBME PSE would be the most valid tool to assess knowledge, despite its limitations. The high correlation found between standardized tests may indicate common factors outside of clerkship experience, which underlie individual performance, such as general medical knowledge and test-taking abilities.

## Conclusions

FCR provides a myriad of benefits to participants, including medical students. The lack of any deleterious association between this practice and knowledge acquisition should provide reassurance to students, faculty, educators and clerkships directors. Future studies should objectively assess the benefit of this experience on student communication, professionalism, and physical examination skills.
